# A cysteine-rich secretory protein involves in phytohormone melatonin mediated plant resistance to CGMMV

**DOI:** 10.1186/s12870-023-04226-7

**Published:** 2023-04-25

**Authors:** Ling-Ling Yang, Qing-Lun Li, Xiao-Yu Han, Xing-Lin Jiang, He Wang, Ya-Juan Shi, Lin-Lin Chen, Hong-Lian Li, Yi-Qing Liu, Xue Yang, Yan Shi

**Affiliations:** 1 College of Plant Protection, Henan Agricultural University, Zhengzhou, 450002 China; 2 Guangdong Baiyun University, Guangzhou, 510550 China

**Keywords:** Melatonin, *Tobamovirus*, *Cucumber green mottle mosaic virus*, Preventive and therapeutic effects, Cysteine-rich secretory protein, CRISP1

## Abstract

**Background:**

Melatonin is considered to be a polyfunctional master regulator in animals and higher plants. Exogenous melatonin inhibits plant infection by multiple diseases; however, the role of melatonin in *Cucumber green mottle mosaic virus* (CGMMV) infection remains unknown.

**Results:**

In this study, we demonstrated that exogenous melatonin treatment can effectively control CGMMV infection. The greatest control effect was achieved by 3 days of root irrigation at a melatonin concentration of 50 μM. Exogenous melatonin showed preventive and therapeutic effects against CGMMV infection at early stage in tobacco and cucumber. We utilized RNA sequencing technology to compare the expression profiles of mock-inoculated, CGMMV-infected, and melatonin+CGMMV-infected tobacco leaves. Defense-related gene *CRISP1* was specifically upregulated in response to melatonin, but not to salicylic acid (SA). Silencing *CRISP1* enhanced the preventive effects of melatonin on CGMMV infection, but had no effect on CGMMV infection. We also found exogenous melatonin has preventive effects against another *Tobamovirus*, *Pepper mild mottle virus* (PMMoV) infection.

**Conclusions:**

Together, these results indicate that exogenous melatonin controls two *Tobamovirus* infections and inhibition of CRISP1 enhanced melatonin control effects against CGMMV infection, which may lead to the development of a novel melatonin treatment for *Tobamovirus* control.

**Supplementary Information:**

The online version contains supplementary material available at 10.1186/s12870-023-04226-7.

## Background

*Cucumber green mottle mosaic virus* (CGMMV) was first found to infect cucumbers in England in 1935 [1], and has since spread worldwide. Following CGMMV infection, plants show mottling and mosaic symptoms on leaves and fruit surfaces, brown necrotic lesions on stem and peduncles, stunted growth, and fruit distortion [2]. CGMMV is a member of the genus *Tobamovirus*, and has a positive single-stranded genomic RNA of approximately 6.4 kb [3]. CGMMV virions are stable and easily transmitted through contact and seed transmission, leading to significant economic losses [2]. At present, methods for the effective control of CGMMV are lacking. Therefore, it is of particular interest to identify effective CGMMV control measures.

Melatonin is considered to be a polyfunctional master regulator in animals and higher plants. Melatonin was first detected as a plant phytohormone in 1995 [4, 5], and its receptor CAND2/PMTR1 was recently discovered in *Arabidopsis thaliana* [6]. Melatonin plays major roles in plant growth and development, and is inducible in response to diverse biotic and abiotic stresses. Melatonin is a crucial protectant [7] that improves plant tolerance to cold [8], drought [9, 10], heat [11], and heavy metal toxicity [12, 13], thus promoting seed germination [14], rooting [15], fruiting [16], and fruit storage [17]. Previous studies have suggested that serotonin, which is the precursor of melatonin, is involved in rice defense responses against *Bipolaris oryzae* infection [18]. Accumulating evidence has shown that exogenous melatonin treatment inhibits infection of plants by viral, fungal, and bacterial pathogens. Exogenous melatonin treatment improves resistance to plant pathogens, through activating antioxidant genes [19], manipulating lignin and gossypol biosynthesis [20], reactive oxygen and nitrogen species scavenging [21, 22], cell wall and callose accumulation [23], hormonal cross-talk [24, 25], PTI (pattern-triggered immunity) and ETI (effector-triggered immunity) regulation [26], and pathogenesis-related protein induction [24, 25]. Compared with fungal and bacterial pathogens, few studies have examined the control of plant viruses through melatonin application. Exogenous melatonin improves rice resistance to *Rice stripe virus* (RSV) through a NO-dependent pathway [27]. Melatonin has also been shown to increase eggplant resistance to *Alfalfa mosaic virus* (AMV) infection [28] and apple resistance to *Apple stem grooving virus* (ASGV) infection [29]. Exogenous melatonin has been shown to improve *Nicotiana glutinosa* and *Solanum lycopersicum* resistance to *Tobacco mosaic virus* (TMV), and to induce the accumulation of salicylic acid (SA), nitric oxide (NO), and the defense-related genes *PR1* and *PR5* [25]. Melatonin control effects for other *Tobamovirus*, including CGMMV, is unclear.

Cysteine-rich secretory protein (CRISP), antigen 5, and pathogenesis-related protein 1 (PR1) form a superfamily of secreted proteins with various functions, that are collectively termed CAP genes [30]. Many mammalian CAP superfamily genes have the potential roles in health and disease. In plants, some CRISPs are involved in plant defense, ginkbilobin2 (Gnk2) has antifungal activity against *Fusarium* spp. [31] and TaCRR1 participates in the defense against both *Rhizoctonia cerealis* and *Bipolaris sorokiniana* in wheat [32]. However, the functions of most CRISPs remain unknown. In previous study, we used CRISP1 in *Nicotiana benthamiana*, to screen genes related to melatonin-induced resistance against CGMMV through transcriptome analysis. The amino acid sequence of CRISP1 showed 98% homology with PR-1 (XP_009759147.1) in *Nicotiana sylvestris*. PRs are a class of defense-related proteins induced by biotic and abiotic stresses in many plant species. PRs have been classified into 17 families [33]. Among these PR1 family are the most abundant plant proteins associated with pathogen attack; therefore, *PR1* gene expression has long been used as a marker for salicylic acid (SA)-mediated disease resistance [34], also can be specificly induced by cytokinins [35], which have a cross-talk with melatonin [36]. PR1 family members were first identified from *Nicotiana tabacum* L. infected with TMV [37]. Plant PR1 family members include both basic and acidic proteins, with no consistent amino acid sequence or function differences between acidic and basic proteins [38]. In tobacco, some acidic PR1 proteins (PR-1a, PR-1b, and PR-1c) are found in extracellular spaces, and can be induced by SA or TMV infection [39, 40]. Basic PR1 proteins, including tomato P14c and tobacco PR-1 g proteins, show the highest antifungal activity [41, 42]. However, many members of the PR1 family remain poorly understood.

In this study, we examined the potential preventative and therapeutic effects of exogenous melatonin treatment on early stage CGMMV infection in tobacco. We used high-throughput sequencing to compare global gene expression in CGMMV infection among wild-type (WT), CGMMV-infected, and melatonin+CGMMV-infected plants. A defense related gene, *CRISP1* was specifically up-regulated in melatonin+CGMMV-infection plants. We also examined the effects of silencing *CRISP1* genes on the CGMMV resistance effects of melatonin. We showed novel functions of CRISP1, which involved in regulation of melatonin in plants. We also found exogenous melatonin could control CGMMV infection in cucumber, and has preventive effects against another *Tobamovirus*, PMMoV infection. This study indicates novel finding that the connection of melatonin and CRISP1 provides a new perspective for further application of the functionally redundant genes on virus control.

## Results

### Exogenous melatonin treatment enhanced *N. benthamiana* resistance to CGMMV

**Fig. 1:**
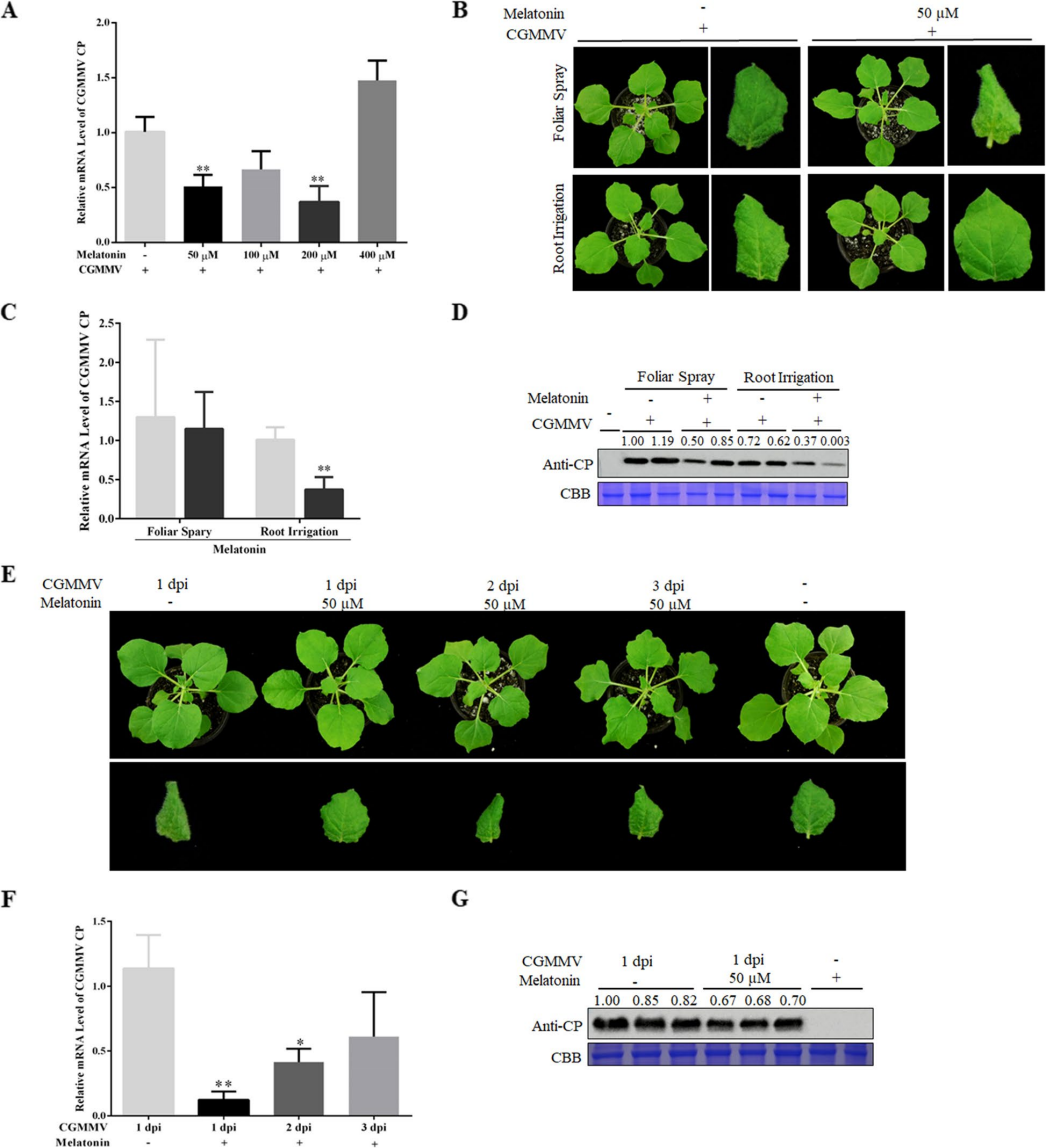
Exogenous melatonin treatment enhanced plant resistance to *Cucumber green mottle mosaic virus* (CGMMV) in tobacco. **A** Quantitative reverse-transcription polymerase chain reaction (qRT-PCR) was performed to examine CGMMV coat protein (CP) expression levels following treatment with 50, 100, 200, and 400 μM melatonin at 9 days post-inoculation (dpi) via root irrigation. **B** Symptoms caused by CGMMV after 3 days of melatonin treatment (foliar spray or root irrigation) at 9 dpi. CGMMV CP accumulation in systemically infected leaves at 9 dpi, determined by (**C**) qRT-PCR and (**D**) Western blotting using CGMMV CP antibody. **E** Symptoms observed at 9 dpi in plants treated with 50 μM melatonin for 3 days via irrigation after inoculation with CGMMV for 1, 2, or 3 days. CGMMV CP accumulation in systemically infected leaves at 9 dpi, determined by (**F**) qRT-PCR and (**G**) Western blotting analysis using CGMMV CP antibody. Bars are standard error of the mean (SEM) from three biological repeats. Significant differences were tested using a two-sample unequal variance *t*-test (*P < 0.05; **P < 0.01). Full-length blots/gels are presented in Supplementary Fig. 4

Accumulating evidence has suggested that melatonin plays important roles in plant defense. In this study, we investigated whether melatonin can effectively suppress CGMMV infection. To examine the effect of phytohormone melatonin on CGMMV infection in *N. benthamiana*, we applied different concentrations of melatonin via root irrigation to treat CGMMV-infected plants. First, we applied 50, 100, 200, and 400 μM melatonin to plants via root irrigation for 3 days and then inoculated with CGMMV. At 9 days post-inoculation (dpi), qRT-PCR was performed to determine CGMMV CP transcript levels in response to melatonin treatment. The results showed that root irrigation containing 50 and 200 μM melatonin suppressed CGMMV CP transcription in systemic leaves. Other concentrations of melatonin had no significant effect on CGMMV accumulation (Fig. 1A). Based on these results, considering practicability and expense, we concluded that 50 μM melatonin is the optimal concentration for suppressing CGMMV infection in *N. benthamiana*.

Next, we explored stable and effective melatonin application methods for the suppression of CGMMV infection. We inoculated plants with CGMMV after 3 days of melatonin treatment via foliar spray or root irrigation. At 9 dpi, curling and chlorosis symptoms were observed in systemic leaves. CGMMV symptoms were less severe in plants treated via root irrigation than foliar spray (Fig. 1B), and lower mRNA and protein levels of CGMMV CP were observed in plants treated via root irrigation than foliar spray, according to qRT-PCR (Fig. 1C) and western blotting (Fig. 1D). Thus, we concluded that root irrigation provided more stable protection against CGMMV infection than foliar spray.

Since melatonin had significant preventive effects against CGMMV infection, we investigated whether melatonin has therapeutic effects for CGMMV infection. We applied 50 μM melatonin via irrigation after plant inoculation with CGMMV for 1, 2, or 3 days; irrigation with water was used as a control. At 9 dpi, no obvious symptoms were observed in melatonin-treated plants that had been inoculated with CGMMV for 1 day, whereas curling symptoms were observed in those inoculated for 2 and 3 days; the control showed more severe symptoms than the latter two groups (Fig. 1E). According to the qRT-PCR results, melatonin treatment after 1 day of CGMMV inoculation had the greatest therapeutic effect (Fig. 1F). Western blotting revealed less CGMMV CP accumulation in systemic leaves of melatonin-treated than control plants following inoculation with CGMMV for 1 day (Fig. 1G). Thus, the greatest therapeutic effect on CGMMV infection was observed and analyses with qRT-PCR and western blotting in plants treated with 50 μM melatonin through root irrigation for 3 days following inoculation with CGMMV for 1 day.

### Exogenous melatonin treatment showed preventive and therapeutic effects for early stage CGMMV infection

**Fig. 2:**
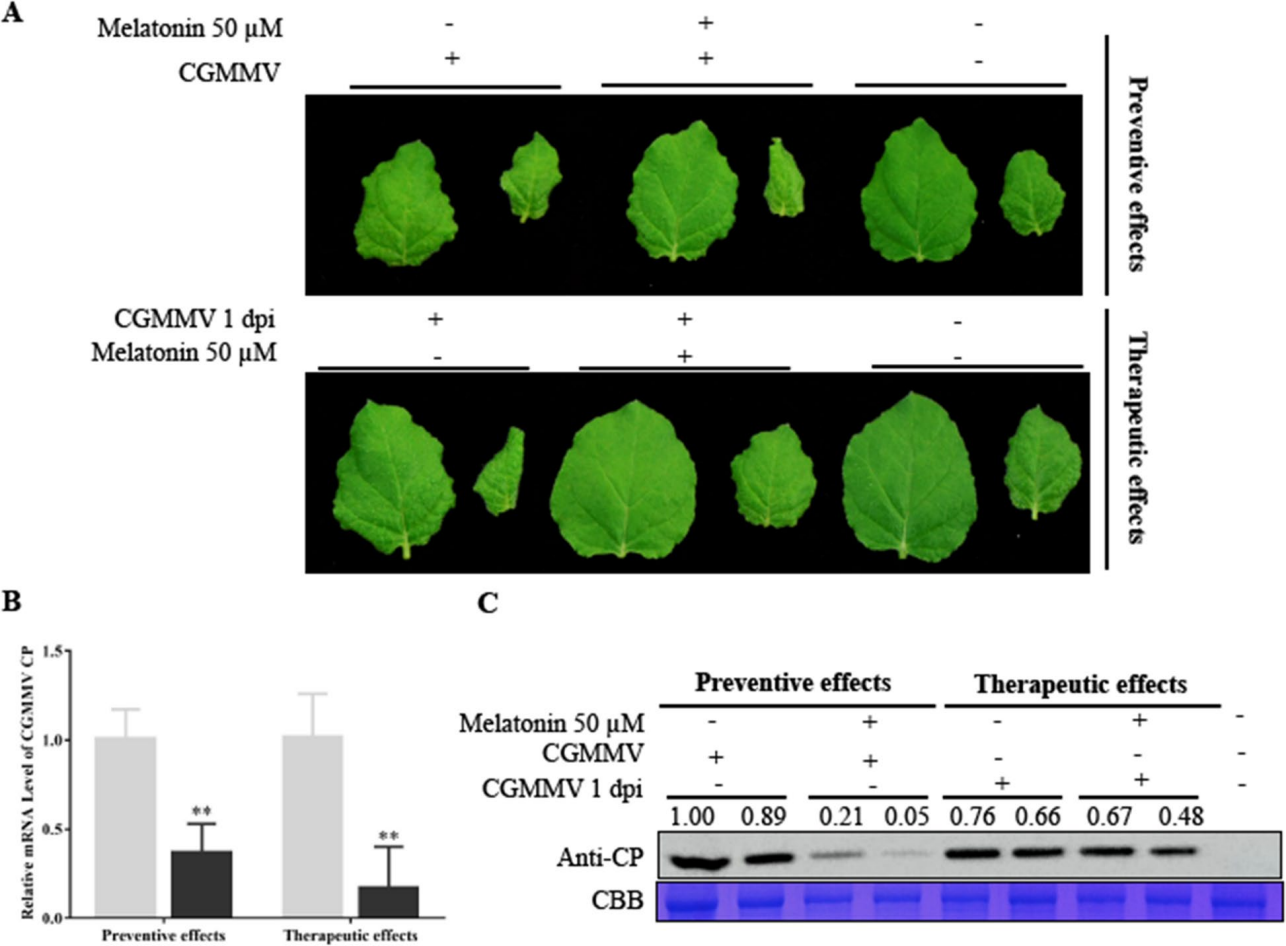
Exogenous melatonin treatment had significant preventive and therapeutic effects for CGMMV infection. **A** Symptoms caused by the effects of melatonin on early stage CGMMV infection at 9 dpi in tobacco. CGMMV CP accumulation in systemically infected leaves at 9 dpi was examined using qRT-PCR (**B**) and Western blotting (**C**) with CGMMV CP antibody. Bars are SEM from three biological repeats. Significant differences were evaluated using a two-sample unequal variance *t*-test (*P < 0.05; **P < 0.01). Full-length blots/gels are presented in Supplementary Fig. 5

Next, we analyzed the preventive and therapeutic effects of melatonin for early stage CGMMV infection. We applied 50 μM melatonin via irrigation for 3 days before and after CGMMV inoculation for 1 day. At 9 dpi, curling and chlorosis symptoms were more severe in systemic leaves of plants untreated with melatonin than in treated plants (Fig. 2A). Melatonin treatment suppressed the accumulation of CGMMV CP according to qRT-PCR (Fig. 2B) and Western blotting (Fig. 2C); however, the preventive effect of melatonin treatment on CGMMV infection was slightly more stable than the therapeutic effect. Together, these results demonstrate that melatonin has both preventive and therapeutic effects for CGMMV infection in *N. benthamiana*.

### Expression profiling and functional categorization of melatonin effects in CGMMV-responsive genes

**Fig. 3:**
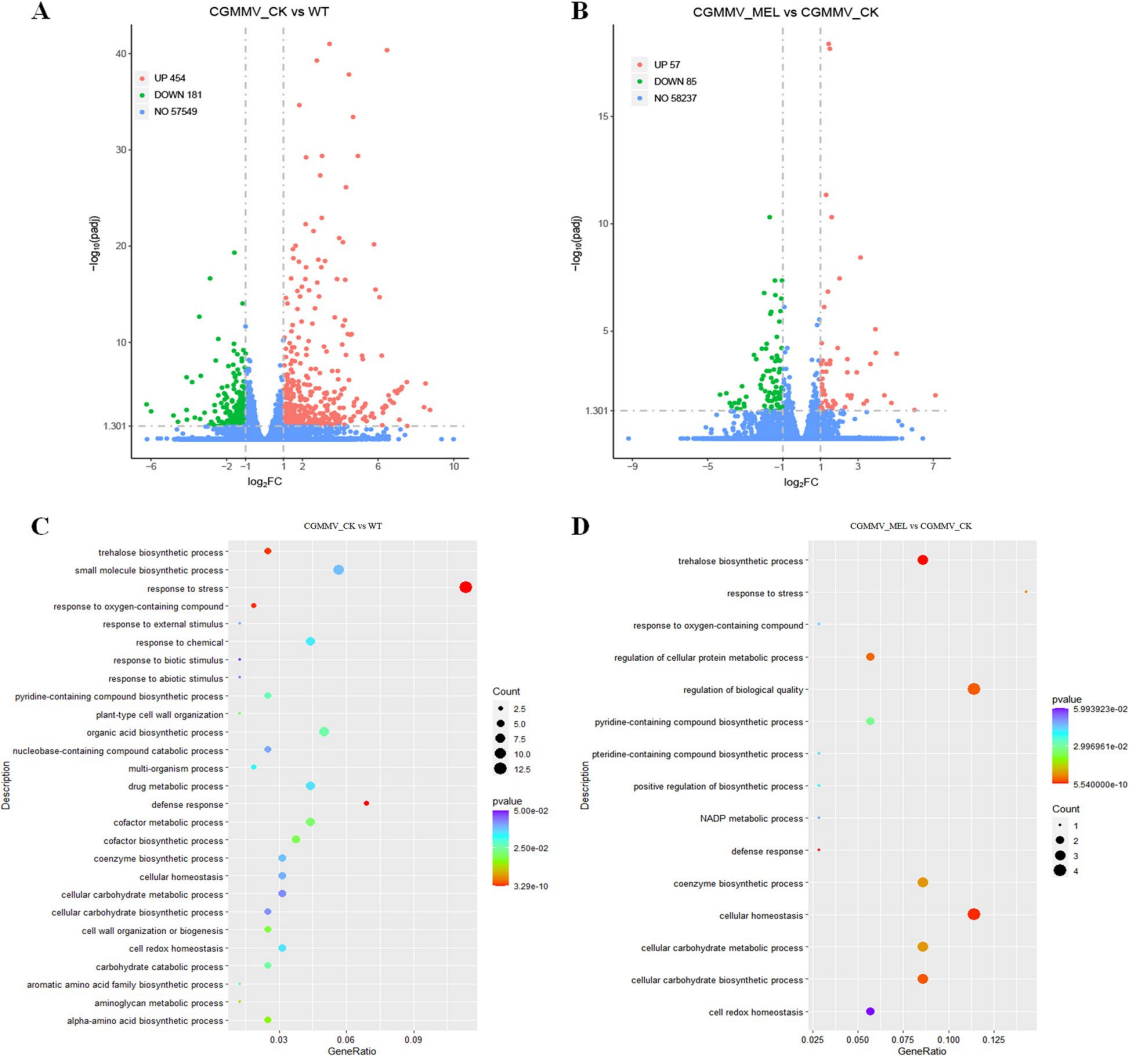
Overview of melatonin-induced CGMMV prevention responses in tobacco transcriptome. Scatterplot of differentially expressed genes (DEGs) in tobacco following (**A**) CGMMV infection (CGMMV_CK vs. WT) and (**B**) melatonin+CGMMV infection (CGMMV_MEL vs. CGMMV_CK). Red and green dots indicate up- and downregulated DEGs, respectively, and blue dots indicate genes that were not significantly affected by treatment. DEGs were evaluated according to the following criteria: false discovery rate ≤ 0.05 and |log_2_(fold change)| ≥ 1. Data represent three biological replicates. Gene Ontology analysis results for DEGs between (**C**) CGMMV_CK and WT, and (**D**) CGMMV_MEL and CGMMV_CK

To further investigate the molecular mechanisms underlying the role of melatonin in plant antiviral defenses, we performed RNA-Seq to compare the expression profiles of three tobacco plant treatments: mock, CGMMV, and melatonin+CGMMV. Here, we used the preventive effect treatment, which is more stable, for melatonin+CGMMV. Preliminary experiments confirmed that mottling and mosaic symptoms appeared on leaves at 9 dpi (Fig. 2); therefore, we analyzed global transcriptomic changes at 9 dpi using leaves collected under each treatment. Total RNA was extracted from three biological replicates, each containing eight plants, for transcriptome analysis. Approximately 42–48 million reads were generated from each RNA-Seq library; most (97%) were mapped to the *N. benthamiana* genome sequence (Fig. S1). A total of 635 differentially expressed genes (DEGs) were identified, with 454 and 181 genes being upregulated and downregulated, respectively, in response to CGMMV infection (CGMMV_CK vs. WT) (Fig. 3A). A total of 142 DEGs were identified, with 57 and 85 genes being upregulated and downregulated, respectively, in response to melatonin+CGMMV inoculation (CGMMV_MEL vs. CGMMV_CK) (Fig. 3B). All DEGs are listed in Tables S2 and S3.

DEGs between the CGMMV_CK vs. WT and CGMMV_MEL vs. CGMMV_CK groups were subjected to Gene Ontology (GO) analysis to predict their involvement in biological processes. Genes affected by CGMMV infection were significantly annotated with 27 GO terms, whereas those affected by melatonin and CGMMV were significantly annotated with 15 GO terms (Fig. 3C and D). Among these, the most significant common GO terms were stress (GO:0006950), trehalose biosynthesis (GO:0005992), and defense response (GO:0006952).

### Expression changes in defense- and stress-related genes in response to CGMMV and melatonin+CGMMV infection

**Fig. 4:**
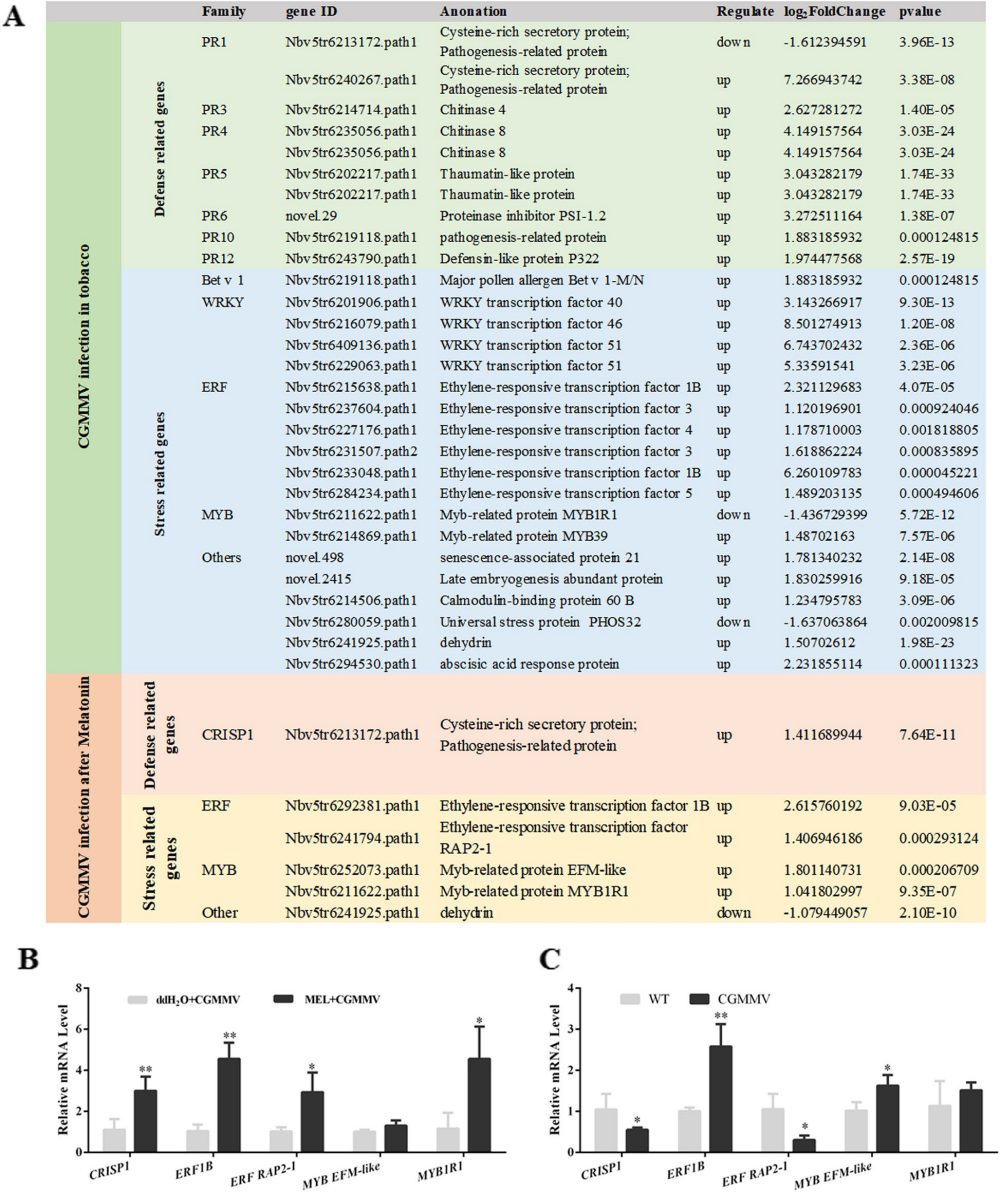
Critical DEGs involved defense- and stress-related genes in response to CGMMV and melatonin+CGMMV infection. **A** Summary of DEGs in defense- and stress-related genes in response to CGMMV and melatonin+CGMMV infection. **B** qRT-PCR was performed to examine the transcript levels of five genes (*CRISP1*, *ERF1B*, *ERF RAP2-1*, *MYB EFM-like*, and *MYB1R1*) in response to melatonin+CGMMV inoculation after melatonin treatment. **C** qRT-PCR was performed to examine the transcript levels of *CRISP1*, *ERF1B*, *ERF RAP2-1*, *MYB EFM-like*, and *MYB1R1* in response to CGMMV infection. Bars are SEM from three biological repeats. Significant differences were evaluated using a two-sample unequal variance *t*-test (*P < 0.05; **P < 0.01)

Many defense- and stress-related genes showed altered expression following CGMMV or melatonin+CGMMV inoculation (Fig. 4A). We identified 11 defense-related and 18 stress-related genes that responded to CGMMV infection. All of these genes were upregulated, except for *CRISP1* (Nbv5tr6213172.path1) and *MYB1R1* (Nbv5tr6211622.path1). Compared with the defense- and stress-related genes that responded to CGMMV infection, only five genes were upregulated only in response to melatonin+CGMMV inoculation: one defense-related gene (*CRISP1*, Nbv5tr6213172.path1) and four stress-related genes including *MYB1R1* (Nbv5tr6211622.path1). Similarly, the qRT-PCR data showed that four defense- and stress-related genes [*CRISP1*, ethylene-responsive transcription factor 1B (*ERF1B*), *ERF RAP2-1*, and *MYB1R1*] had higher expression levels under melatonin+CGMMV treatment (Fig. 4B). These results indicate the activation of tobacco defense and stress responses, which may play an important role in CGMMV infection, and that melatonin treatment prior to CGMMV infection specifically activated *CRISP1*, *ERF1B*, *ERF RAP2-1*, and *MYB1R1* expression.

We also performed qRT-PCR to detect the transcript levels of these genes *CRISP1*, *ERF1B*, *ERF RAP2-1*, and *MYB1R1* in response to melatonin prior to CGMMV inoculation (Fig. 4B) and only CGMMV infection (Fig. 4C). The results showed significantly decreased *CRISP1* and *ERF RAP2-1* transcript levels in response to CGMMV infection (Fig. 4C). *MYB1R1* showed no expression change according to qRT-PCR, whereas the transcriptome data showed downregulated *MYB1R1* expression in response to CGMMV infection (Fig. 4A). The results showed that melatonin specifically induces the expression of *CRISP1*, *ERF RAP2-1*, and *MYB1R1*, which were downregulated in response to CGMMV infection. Here, we focused on the function of the defense related gene, *CRISP1*, which was induced melatonin treatment.

### Silencing CRISP1 enhanced the preventive effects against CGMMV infection of melatonin

**Fig. 5:**
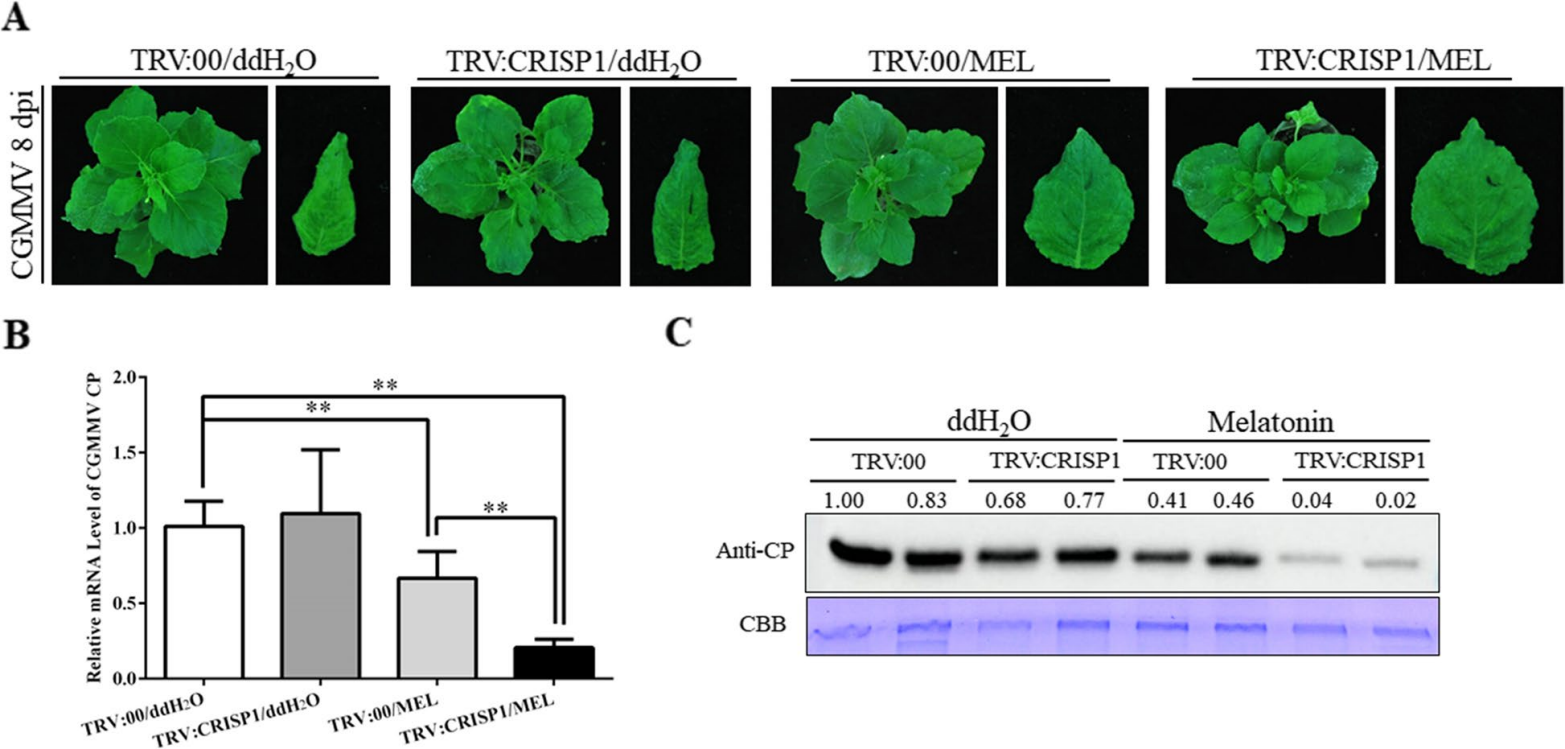
CRISP1 silencing enhanced melatonin-induced CGMMV prevention responses. **A** Systemic infection by CGMMV at 8 dpi in plants pre-treated with TRV:00 or TRV:CRISP1. TRV:00/MEL and TRV:CRISP1/MEL plants were irrigated continuously with melatonin for 3 days prior to CGMMV infection; ddH_2_O was used as a control. CGMMV CP accumulation in systemically infected leaves at 9 dpi was examined using (**B**) qRT-PCR and (**C**) western blotting with CGMMV CP antibody. Bars are SEM from three biological repeats. Significant differences were evaluated using a two-sample unequal variance *t*-test (*P < 0.05; **P < 0.01). Full-length blots/gels are presented in Supplementary Fig. 6

First, we used the *Tobacco rattle virus* (TRV) -based virus-induced gene silencing (VIGS) system to silence CRISP1 in *N. benthamiana* plants. At 7 dpi with TRV:CRISP1 or TRV:00, we irrigated plants with melatonin for 3 days. CGMMV was then mechanically inoculated to TRV:00/ddH_2_O, TRV:CRISP1/ddH_2_O, TRV:00/melatonin (TRV:00/MEL), and TRV:CRISP1/melatonin (TRV:CRISP1/MEL) plants at VIGS 10 dpi. According to the qRT-PCR results, the silencing efficiency of *CRISP1* was approximately 65%, and was unaffected by exogenous melatonin treatment (Fig. S2). The viral infection spread to the topmost leaves, with systemic leaves showing curling and chlorosis symptoms at 8 dpi. These symptoms were slightly more severe in the upper leaves of TRV:CRISP1/ddH_2_O plants than in those of TRV:00/ddH_2_O plants (Fig. 5A). The qRT-PCR and western blotting results showed no difference in CGMMV CP mRNA or protein between TRV:CRISP1/ddH_2_O and TRV:00/ddH_2_O plants (Fig. 5B and C). In melatonin-treated plants, curling and chlorosis symptoms were milder in systemic leaves of TRV:00/MEL and TRV:CRISP1/MEL plants than in those of TRV:CRISP1/ddH_2_O and TRV:00/ddH_2_O plants at 8 dpi (Fig. 5A). Both CGMMV CP mRNA and protein accumulation levels were lower in TRV:00/MEL and TRV:CRISP1/MEL plants than in those of TRV:CRISP1/ddH_2_O and TRV:00/ddH_2_O plants (Fig. 5B and C). These results showed that exogenous melatonin treatment had significant preventive effects against CGMMV infection in both TRV:00 and TRV:CRISP1 infected plants. CGMMV CP mRNA (by 46%) and protein accumulation levels (by 93%) were significantly downregulated in TRV:CRISP1/MEL plants compared with TRV:00/MEL plants (Fig. 5B and C) indicating that CRISP1 silencing enhanced the preventive effects of melatonin against CGMMV infection.

### Transcript levels of *CRISP1* is specifically induced by melatonin, but not by SA or CGMMV

*CRISP1* was not induced by CGMMV, actually the transcript level of *CRISP1* was decreased 1.6-fold in transcriptome data (Fig. 4A) and 2.1-fold in qRT-PCR results (Fig. 4C). *CRISP1* was not a typical defense-related gene for CGMMV. Here, to investigate the function of CRISP1 in response to CGMMV infection or melatonin, we used PR1a (Nbv5tr6240267.path1) as a control, as it exhibits the highest homology with acidic PR-1a (X12737, 41]. Our transcriptome data showed that *PR1a* expression was increased 7.2-fold by CGMMV infection (Fig. 4A), and qRT-PCR showed that *PR1a* expression was increased 25-fold by CGMMV infection (Fig. S3A). *PR1a* (Nbv5tr6240267.path1) was a typical defense-related genes, which has the role in plants’ resistance system against CGMMV. These results indicated that unlike *PR1a*, *CRISP1* was not involve in plants’ resistance system against CGMMV.

**Fig. 6:**
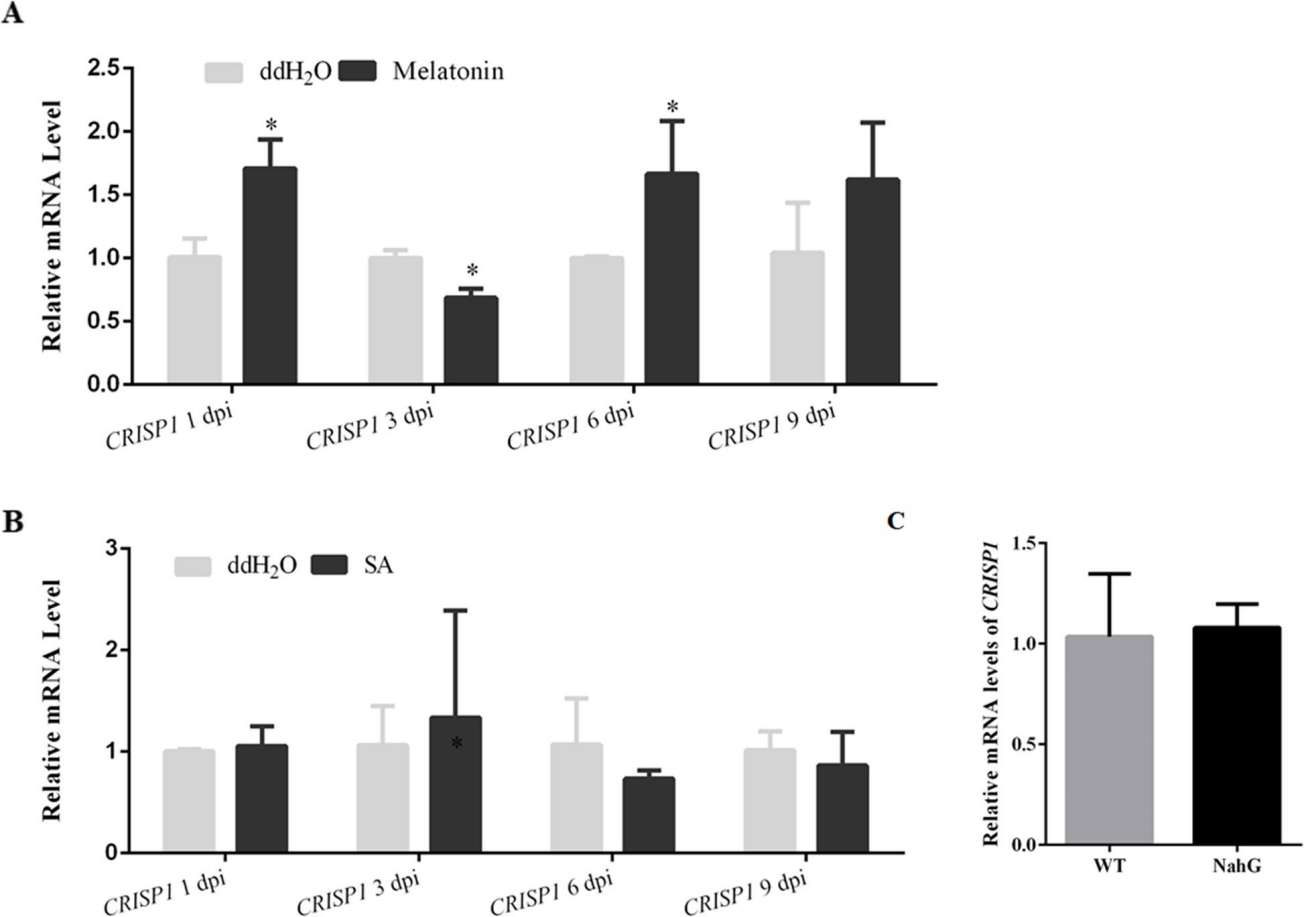
Expression level of CRISP1 in response to melatonin and SA treatment. **A** qRT-PCR was performed to examine *CRISP1* transcript levels in response to melatonin treatment. **B** qRT-PCR was performed to examine *CRISP1* transcript levels in response to SA treatment. **C** qRT-PCR was performed to examine *CRISP1* transcript levels in WT and NahG transgenic plants. Bars are SEM from three biological repeats. Significant differences were evaluated using a two-sample unequal variance *t*-test (*P < 0.05; **P < 0.01)

To further investigate the relationship between melatonin and CRISP1, we irrigated tobacco plants with melatonin for 3 days and performed qRT-PCR analysis of *CRISP1* transcript levels in leaves sampled at 1, 3, 6, and 9 dpi. The results showed that *CRISP1* expression was upregulated by melatonin at 1 and 6 dpi (Fig. 6A). Melatonin treatment had no effect on the induction of *PR1a* expression by CGMMV, consistent with the transcriptome results (Fig. S3B and Fig. 4A). We also investigated whether *PR1a* was induced by melatonin without CGMMV infection. The results showed that *PR1a* expression was decreased at 3, 6, and 9 dpi in response to melatonin treatment (Fig. S3D). These results support the specific induction of *CRISP1* expression by melatonin.

Considering salicylic acid (SA) was associated with the induction of PRs proteins and the establishment of systemic acquired resistance [43], we also investigated the relationship between *CRISP1* and SA. We treated plant leaves with SA for 3 days and performed qRT-PCR analysis of *CRISP1* transcript levels in leaves sampled at 1, 3, 6, and 9 dpi. The results showed that SA did not affect *CRISP1* expression (Fig. 6B). Transgenic plants expressing the bacterial enzyme salicylate hydroxylase (NahG), which degrades SA, have increased susceptibility to many plant pathogens, including viruses [44]. We also detected the *CRISP1* expression in NahG transgenic *N. benthamiana* plants, which has no difference between NahG transgenic *N. benthamiana* plants and wild type *N. benthamiana* (Fig. 6C). Interestingly, *PR1a* was only detected at 1 day after SA treatment, and expression of *PR1a* was up regulated. *PR1a* was undetectable in NahG transgenic *N. benthamiana* plants. These results demonstrated that *CRISP1* was not specifically induced by SA like *PR1a*.

Together, these results demonstrated that *CRIPS1* was specifically induced by melatonin, but not by SA or CGMMV.

### Exogenous melatonin treatment decreases CGMMV accumulation in other host and has effect on other *Tobamovirus*

**Fig. 7:**
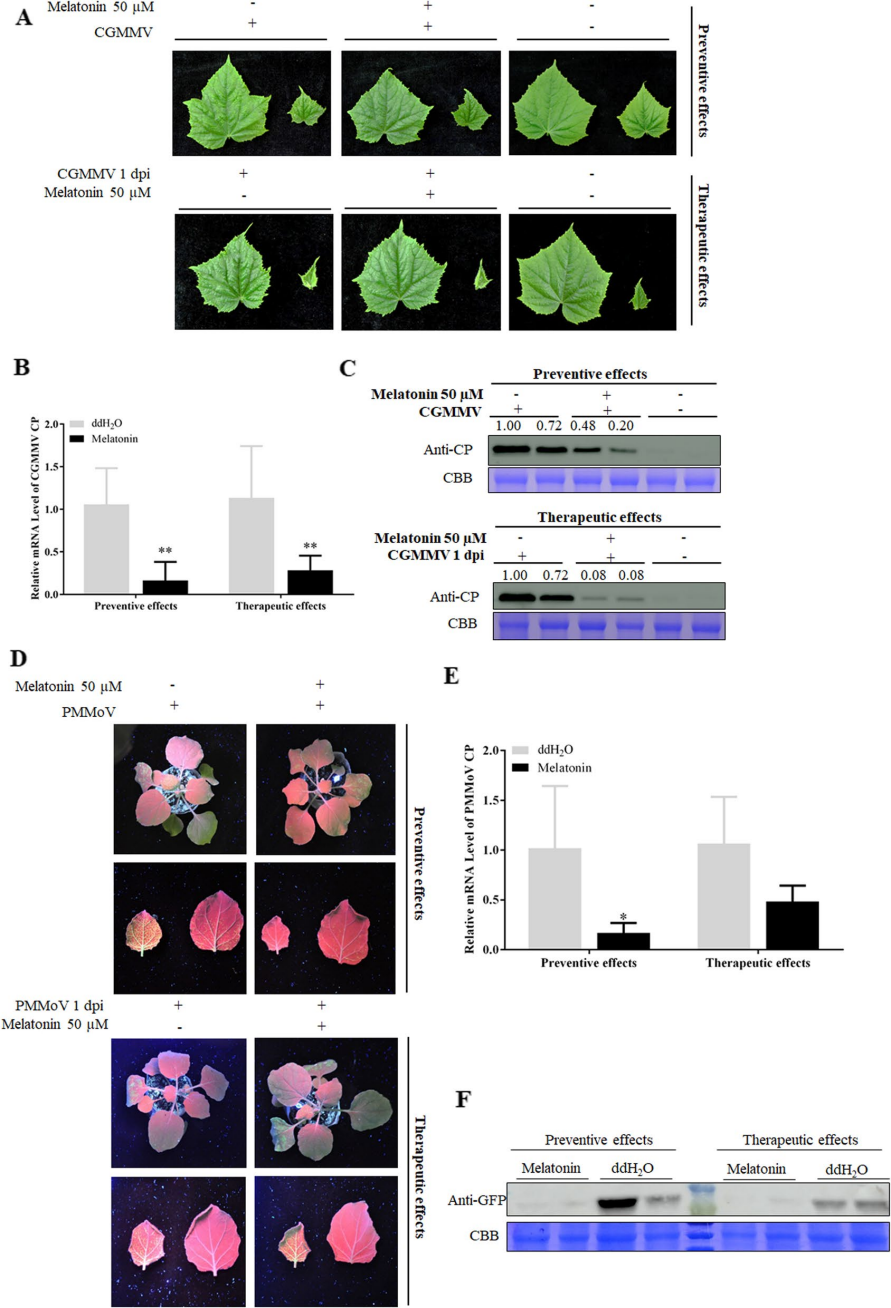
Exogenous melatonin treatment decreases CGMMV accumulation in cucumber and has preventive effect on other *Tobamovirus*. **A** Symptoms of early stage CGMMV infection at 17 dpi on cucumber leaves. CGMMV CP accumulation in systemically infected cucumber leaves at 17 dpi was examined using qRT-PCR (**B**) and Western blotting (**C**) analysis with CGMMV CP antibody. Full-length blots/gels are presented in Supplementary Fig. 7. **D** PMMoV-GFP infection at 7 dpi in tobacco leaves treated by melatonin. PMMoV accumulation in systemically infected leaves at 7 dpi was examined using qRT-PCR (**E**) and western blotting (**F**) analysis with GFP antibody. Full-length blots/gels are presented in Supplementary Fig. 8. Bars are SEM from three biological repeats. Significant differences were evaluated using a two-sample unequal variance *t*-test (*P < 0.05; **P < 0.01)

CGMMV causes significant yield and quality losses in many cucurbit species. In this study, we also used cucumber (*Cucumis sativus* L.), which is the natural host of CGMMV, to test the preventive and therapeutic effects of melatonin application on CGMMV. We treated plants with 50 μM melatonin via irrigation for 3 days, prior to and 1 day after CGMMV inoculation. At 17 dpi, green mottling occurred on true leaves of control plants untreated with melatonin, whereas those of plants treated with melatonin showed delayed and milder symptoms (Fig. 7A). QRT-PCR and western blotting showed that melatonin treatment suppressed CGMMV CP accumulation (Fig. 7B, C). Together, the results demonstrate that melatonin treatment effectively controlled CGMMV infection in cucumber.

Exogenous melatonin treatment effectively controlled CGMMV infection, we wondered whether melatonin treatment control another virus. Here, we used the infection clone of PMMoV, which is a member of the genus *Tobamovirus* too, to infect tobacco leaves after or before melatonin treatment to test the control effects of melatonin. The melatonin application methods for PMMoV were the same as that for CGMMV. At 7 dpi, the plants were further challenged with a modified clone of PMMoV expressing the green fluorescent protein (PMMoV-GFP) and monitored for symptom development. Newly emerging leaves show curling symptoms and green fluorescence under UV illumination. The green fluorescence appearing on most of the top leaves of melatonin treated plants was weaker than that in the control plants umder the 50 μM melatonin treatment prior to PMMoV-GFP infection (Fig. 7D, upper panels). But the accumulation of green fluorescence on the top leaves of melatonin treated leaves was the same as the control plants on the treatment melatonin 1 days after PMMoV-GFP infection (Fig. 7D, bottom panels). QRT-PCR results indicated that melatonin showed preventive effects against PMMoV-GFP infection without influencing therapeutic effects (Fig. 7E). Western blotting showed that the PMMoV-GFP accumulation in the systemic leaves was significantly lower in both melatonin treatments compared to the controls (Fig. 7F). Together, the results demonstrate that melatonin treatment effectively controlled another *Tobamovirus*, PMMoV infection.

## Discussion

Melatonin has multiple regulatory effects on both biotic and abiotic stress in plants. Exogenous melatonin treatment can inhibit viral, fungal, and bacterial pathogens infection in plants. Plant viruses inhibited by melatonin treatment include TMV [25], RSV [27], AMV [28], and ASGV [29]. Exogenous melatonin treatment induces systemic signals promoting plant defenses against pathogens, and therefore has potential as a pathogen control method [25]. However, the effects of melatonin on CGMMV remain unknown. In this study, we found that melatonin application had both preventive and therapeutic effects for CGMMV infection in tobacco. Foliar spray and root irrigation are two effective melatonin application methods for the control of plant pathogens [45]. In this study, root irrigation was a more stable and effective method than foliar spray for melatonin application to control CGMMV infection, possibly because melatonin is absorbed mainly through the roots [46]. Root irrigation has been used for melatonin treatment to confer plant resistance to TMV [25]. The melatonin concentration used for pathogen control typically ranges from 1 μM to 10,000 μM [45]. In this study, we used 50, 100, 200, and 400 μM melatonin to control CGMMV infection and found that 50 μM was the optimal concentration; all of the other treatments failed to effectively prevent CGMMV infection, consistent with previous studies of TMV [25] and RSV [27]. Melatonin significantly reduced the accumulation of RSV at a concentration of 10 μM, but not 100 μM [27]. Treatment with 100 μM melatonin via root irrigation resulted in reduced TMV titers in *N. glutinosa* and *S. lycopersicum*, whereas concentrations of 200 and 400 μM had no significant effect on SA levels or TMV control [25]. We speculate that high melatonin concentrations inhibit melatonin-induced plant defense against viruses, and may promote viral infection via unknown pathways. Previous studies have demonstrated that exogenous melatonin application can promote glutathione-dependent induction of local and systemic defenses against oxidative stress in cucumber [47] and boost resistance against the foliar pathogen *Podosphaera xanthii* (powdery mildew) and soil-borne oomycete *Phytophthora capsici* in watermelon, cucumber, and other cucurbits [26]. Meanwhile, we found melatonin treatment can effectively control CGMMV accumulation in cucumber. These findings indicate that increasing melatonin levels in specialty crops such as cucumber, watermelon, and other cucurbits could improve local and systemic defenses against plant pathogens. We also found melatonin treatment have not only the affective for CGMMV and TMV [25], but also for another *Tobamovirus* PMMoV.

Melatonin enhances pathogen resistance by inducing the expression of a number of plant defense- and stress-related genes. In this study, we found that three genes (*CRISP1*, *ERF RAP2-1*, and *MYB1R1*) were specifically induced by melatonin. Among these genes, *CRISP1* was a predicted defense-related gene. CRISP1 is a CAP superfamily member that has 98% homology with PR-1 (XP_009759147.1) in *N. sylvestris*. We speculate that CRISP1 is a PR1-like protein. In previous researches, a PR-10-fold protein from *Hypericum perforatum* [48] and yellow lupine (*Lupinus luteus*) [49], was crystallized in complex with melatonin (MEL). These researches implicated at the high melatonin concentration the PR-10 proteins act as low-affinity melatonin binders and PR family members actually had realition with melatonin. Recently, Guo and colleges [50] found melatonin biosynthetic rate-limiting enzyme N-acetylserotonin O-methyltransferase 2 (MeASMT2) in cassava physically interacted with MePR1 to promote anti-bacterial activity against to cassava bacterial blight. In this study, transcripts of MePR1 was increased in response to *Xam* infection. However, *CRISP1* is not induced by pathogens or SA, whereas PR1a, an acidic defense-related protein, show significant induction by CGMMV infection and SA in plants in this study. In tobacco, some acidic PR1 proteins (PR-1a, PR-1b, and PR-1c) have been induced by SA and TMV infection [39, 40]. In our study, *CRISP1* is stably expressed in wild type or NahG transgenic *N. benthamiana* plants, whereas *PR1a* was undetectable in NahG transgenic *N. benthamiana* plants. These findings indicate that *CRISP1* was a constitutive expression in plant in response to melatonin, while *PR1a* was an inducible expression gene in response to CGMMV and SA, not by melatonin. Pervious research showed melatonin elicited defense signaling pathway though SA, which could up regulated *PR* genes [51]. In our study, exogenous melatonin induced the expression of PR1-like protein, CRISP1, instead of PR1a. However, *CRISP1* is not induced by SA. We speculated that *CRISP1* was a specific downstream regulator by melatonin. CRISP1 may be functionally redundant with respect to CGMMV resistance in plants, and melatonin specifically induced CRISP1. These results deepen our understanding of CRIPS1 in plants.

*CRISP1* was not induced by CGMMV infection or SA, and its silencing did not affect CGMMV-infected plants; therefore, CRISP1 may not be directly involved in plant defenses against CGMMV infection. *CRISP1* silencing enhanced the preventive effects of melatonin against CGMMV infection. Thus, CRISP1 may be a negative factor with respect to melatonin induction, and may inhibit the melatonin-mediated defense against CGMMV. CRISP1 may also play a role in the balance between plant defense and plant growth and development in the context of the melatonin-mediated defense against plant pathogens.

## Conclusions

In summary, we report that exogenous melatonin application enhanced tobacco and cucumber resistance to CGMMV, with both preventive and therapeutic effects seen at an optimal concentration of 50 μM achieved via root irrigation. Melatonin also could effectively control another *Tobamovirus* PMMoV infection in tabacoo. We screened three genes specifically induced by melatonin (*CRISP1*, *ERF RAP2-1*, and *MYB1R1*) using high-throughput sequencing. *CRISP1* silencing enhanced the control of CGMMV by melatonin, but had no effect on CGMMV infection or SA treatment. These results suggest that CRISP1 was a specific downstream regulator by melatonin and knocking out certain functionally redundant genes may improve the effectiveness of melatonin (and other chemicals) for controlling plant pathogen outbreaks.

## Methods

### Plant materials and CGMMV inoculation

Wide type *Nicotiana benthamiana*, *NahG* transgenic *N. benthamiana* plants (provided by Dr. Yule Liu, Tsinghua University, Beijing, China) and *Cucumis sativus* L. were grown under a 16-h light/8-h dark regime at 25°C. CGMMV, which from CGMMV infected leaves homogenate suspension with water, was inoculated mechanically onto tobacco at five-leaf-satgeand cucumber seedlings leaves using the classical method, and *Agrobacterium* harboring an infectious clone of CGMMV [52] was used for infiltration. And the infectious clone of *Pepper mild mottle virus* (PMMoV, provided by Dr. Fei Yan, Ningbo University, Zhejiang, China) [53] was used for infiltration to tobacco leaves.

### Melatonin and SA treatments

Melatonin was applied through either foliar spray or root irrigation [45]. *Nicotiana benthamiana* plants were sprayed or irrigated daily with melatonin (M5250; Sigma-Aldrich, St. Louis, MO, USA) at different concentrations (50, 100, 200, or 400 μM) dissolved with ddH_2_O for 3 days; ddH_2_O was used as a control. On day 4, CGMMV was inoculated onto plant leaves. Preventive treatment consisted of daily irrigation of *N. benthamiana* and *C. sativus* plants with 50 μM melatonin for 3 days, followed by inoculation with CGMMV. Therapeutic treatment consisted of daily irrigation with 50 μM melatonin daily for 3 days after 1, 2, or 3 days of inoculation with CGMMV. Other plants were sprayed daily with 1 mM SA dissolved in 0.1% (v/v) ethanol for 3 days. Mock control plants were treated with 0.1% ethanol. SA was sprayed on both the adaxial and abaxial sides of leaves until runoff occurred. All treatments were performed at least three times independently; at least six sets of consistent data were used for further analyses.

### Quantitative reverse-transcription polymerase chain reaction (qRT-PCR)

Total RNA was isolated from *N. benthamiana* and *C. sativus* leaves using RNAiso Plus (TaKaRa, Shiga, Japan). Genomic DNA was removed from purified total RNA using the RNase-free DNase I gDNA wiper (Vazyme, Nanjing, China). The first-strand cDNA was synthesized using HiScript III RT SuperMix for qPCR (Vazyme, Nanjing, China) according to the manufacturer’s protocol. QRT-PCR was performed to measure CGMMV accumulation using the CGMMV coat protein (CP) primers listed in Table S1, and the expression and silencing efficiency of *CRISP1* (Nbv5tr6213172.path1/Niben101Scf23606g00005.1), *ERF1B* (Nbv5tr6292381.path1/Niben101Scf08965g00003.1), *ERF RAP2-1* (Nbv5tr6241794.path1/Niben101Scf03223g00002.1), *MYB EFM-like* (Nbv5tr6252073.path1/Niben101Scf03080g01015.1), and *MYB1R1* (Nbv5tr6211622.path1/Niben101Scf07437g01012.1) using the primers listed in Table S1. For qRT-PCR, the internal reference gene of *N. benthamiana* was ubiquitin C (*NbUBC*, AB026056.1) [54] and those of *C. sativus* were ubiquitin extension protein (*CsUBI-ep*, AY372537) and elongation factor 1-α (*CsEF1α*, *EF446145*) [55]; the related primers are listed in Table S1. The QuantStudio 3 Real-Time PCR System was used for the reaction, and the results were analyzed using the 2^−ΔΔCT^ method [56]. All treatments were performed at least three times independently; at least six sets of consistent data were used for further analyses.

### Western blotting

Total proteins were extracted from *N. benthamiana* and *C. sativus* leaves using lysis buffer (100 mM Tris-HCl, pH 8.8; 6% sodium dodecyl sulfate, 2% β-mercaptoethanol). Proteins were separated in 15% SDS–polyacrylamide gel electrophoresis (PAGE) gels and incubated with primary and HRP-conjugated secondary antibodies (Transgene Biotech, Beijing, China). After incubation with secondary antibody, detection signals using the EasySee Western Blot Kit (Transgene Biotech, Beijing, China) and imaged using an Amersham Imager 680 (GE Healthcare Life Sciences, Piscataway, NJ, USA). The primary antibodies used in this study were anti-CGMMV CP, prepared in our laboratory. The large subunit of RuBisCo served as a loading control using Coomassie Brilliant Blue (CBB) staining. Quantitative calculation of digital blot images was performed using ImageJ software (NIH, Bethesda, MD, USA).

### RNA sequencing RNA-seq and data analysis

*N. benthamiana* plants were irrigated daily with 50 μM melatonin for 3 days; ddH_2_O was used as a control. On day 4, CGMMV was inoculated onto plant leaves. Leaves were collected for RNA-Seq analysis at 9 dpi. Three replicate leaf samples were collected for each treatment (CGMMV_MEL, CGMMV_CK, and WT) and each sample contained leaves from at least eight plants. At 7 dpi with CGMMV, tobacco leaves were collected for total RNA extraction. The cDNA libraries were constructed using the NEB Next Ultra RNA Library Prep Kit for Illumina (no. 7530 L; New England Biolabs, Ipswich, MA, USA), and the DNA yield and fragment insert size distribution of each library were determined using the Agilent Bioanalyzer 2100 system. The library was sequenced using an Illumina NovaSeq platform following a series of preparatory procedures; 150 bp of paired-end reads were generated, and clean reads were assembled into transcripts using the Hisat2 v2.0.5 tool, with the *N. benthamiana* genome as a reference (http://sefapps02.qut.edu.au/downloads/Nbv0.5.genome.fa.gz). Unigene expression levels were quantified in terms of fragment per kilobase of transcript per million mapped reads (RPKM) and DEGs were screened according to the following criteria: false discovery rate *P* < 0.05 and absolute log_2_ (fold change) ≥ 1. GO enrichment analysis of DEGs was implemented using the clusterProfiler package in R software (R Development Core Team, Vienna, Austria), which corrected gene length bias. GO terms with corrected *P* < 0.05 were considered significantly enriched in DEGs.

### Virus-induced gene silencing (VIGS) assay

TRV vectors were introduced into *Agrobacterium tumefaciens* strain GV3101 [57]. *Agrobacterium-*harboring vectors derived from TRV1 or TRV2 were resuspended in infiltration buffer diluted to an OD_600_ of 1.0 and mixed at a 1:1 ratio. After 4 h of incubation at room temperature, the mixed cultures were infiltrated into young leaves of 5-6 week-old *N. benthamiana plants.* pTRV2 was used to silence host genes by expressing the partial sequence of different plant genes. To silence *N. benthamiana* genes, a 200-300 bp fragment of *CRISP1* mRNA was inserted into the pTRV2 vector, and used an empty pTRV2 vector as the control plants (TRV:00). The primers used for these constructions are listed in Table S1. Silenced and control plants at 10 dpi were used for further analyses.

### Quantification and statistical analysis

Data are reported as means ± standard deviation (SD), calculated using GraphPad Prism software (GraphPad Software Inc., La Jolla, CA, USA). The statistical significance of qRT-PCR results was evaluated using Student’s *t*-test (*P < 0.05; **P < 0.01).

## Supplementary Information

**Additional file 1: Figure S1.** Transcriptome sequencing quality. **Figure S2.** Silence efficiency of *CRISP1* at VIGS 10 dpi. **Figure S3.** Expression level of PR1a in response to CGMMV and melatonin treatment. **Figure S4.** Original picture for Fig.1. **Figure S5.** Original picture for Fig.2. **Figure S6.** Original picture for Fig.5. **Figure S7.** Original picture for Fig.7C. **Figure S8.** Original picture for Fig.7F. 

**Additional file 2: Table S1.** The primers used in this study. **Table S2.** CGMMV_CK vs WT responsive genes in Tobacco. **Table S3.** CGMMV_MEL vs CGMMV_CK responsive genes in Tobacco.

## Data Availability

The datasets generated during the current study are available in the GEO repository, our data accession number is GSE221904.
