# Supplementary material for: A cysteine-rich secretory protein involves in phytohormone melatonin mediated plant resistance to CGMMV
Source: BMC Plant Biol. 2023 Apr 25;23:215. doi: 10.1186/s12870-023-04226-7 (PMC10127030; doi:10.1186/s12870-023-04226-7)
Supplement: Supplementary file 1 — Additional file 1: Figure S1. Transcriptome sequencing quality. Figure S2. Silence efficiency of CRISP1 at VIGS 10 dpi. Figure S3. Expression level of PR1a in response to CGMMV and melatonin treatment. Figure S4. Original picture for Fig.1. Figure S5. Original picture for Fig.2. Figure S6. Original picture for Fig.5. Figure S7. Original picture for Fig.7C. Figure S8. Original picture for Fig.7F. [file 12870_2023_4226_MOESM1_ESM.pdf]

Treatment	Raw reads	Clean reads	Clean bases	Error rate	Q20 (%)	Q30 (%)	GC%	Mapped reads
WT1	45545592	44530332	6.68G	0.03	97.91	94.04	43.23	43324806(97.29%)
WT2	45408444	44076668	6.61G	0.02	97.98	94.23	43	42870211(97.26%)
WT3	43791022	42588408	6.39G	0.03	97.86	93.91	43.15	41447406(97.32%)
CGMMV_CK_1	45846472	44643954	6.7G	0.03	97.87	93.91	42.92	43400695(97.22%)
CGMMV_CK_2	45558346	44371050	6.66G	0.03	97.65	93.28	43	43157292(97.26%)
CGMMV_CK_3	49455838	48149838	7.22G	0.02	98.13	94.55	43.03	46952432(97.51%)
CGMMV_MEL_1	44655646	43451392	6.52G	0.02	97.98	94.21	43.11	42307099(97.37%)
CGMMV_MEL_2	45922690	44779390	6.72G	0.03	97.93	94.06	43.05	43565166(97.29%)
CGMMV_MEL_3	46845744	45583416	6.84G	0.03	97.97	94.18	43.25	44355510(97.31%)

Fig. S1. Transcriptome sequencing quality.

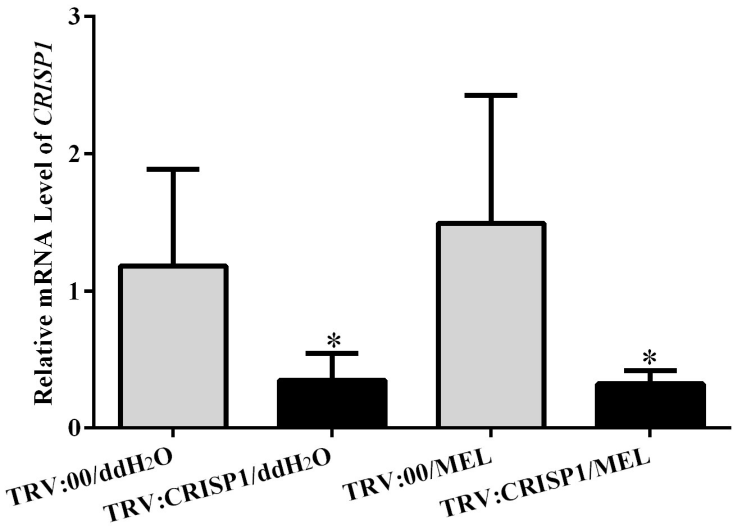

Fig. S2. Silence efficiency of *CRISP1* at VIGS 10 dpi.

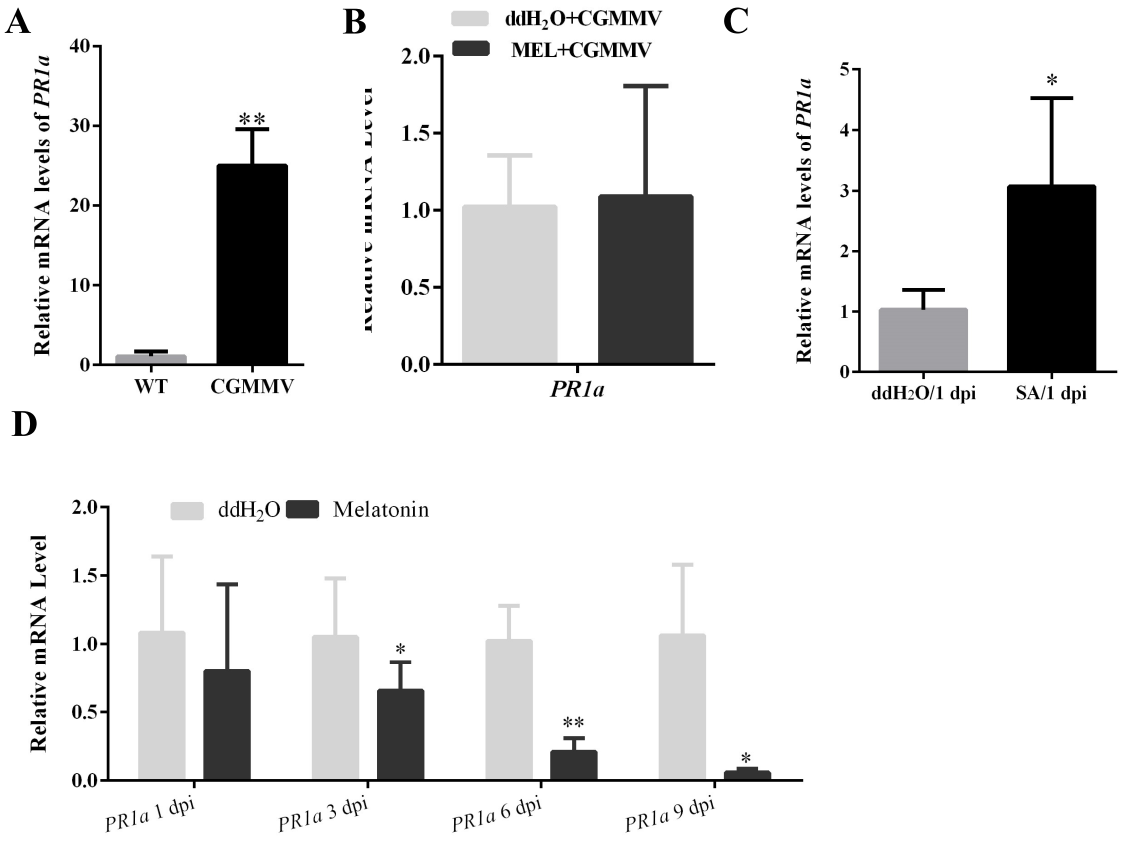

Fig. S3. Expression level of *PR1a* in response to CGMMV and melatonin treatment. (A) *PR1a* accumulation in CGMMV systemically infected leaves at 7 dpi was examined using qRT-PCR. (B) *PR1a* transcript level by CGMMV at 7dpi in plant pre-treated with melatonin for 3 days prior to CGMMV infection determined by qRT-PCR; ddH_2O was used as a control. (C) qRT-PCR was performed to examine *PR1a* transcript levels in response to melatonin treatment. Bars are SEM from three biological repeats. Significant differences were evaluated using a two-sample unequal variance *t*-test (*$P < 0.05$; **$P < 0.01$).

Original picture for Fig.1

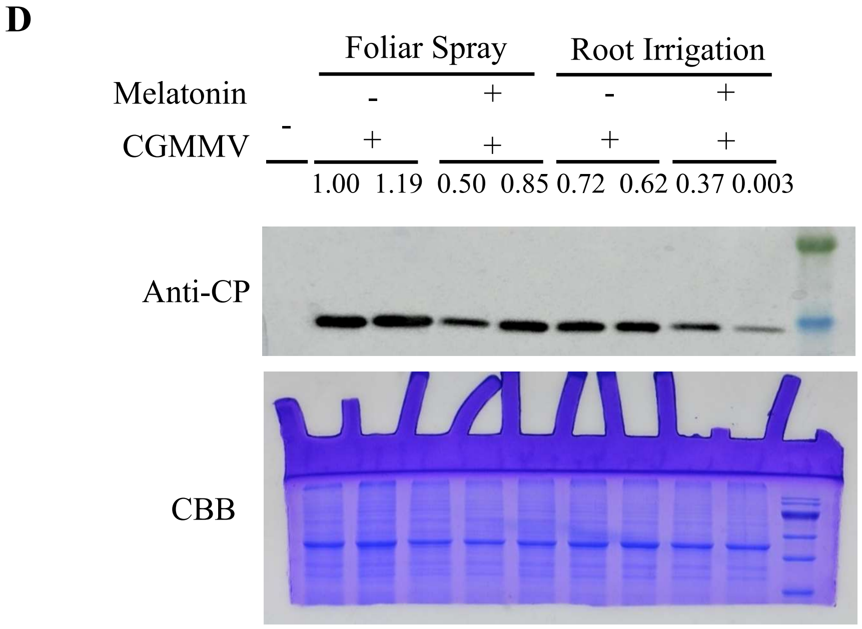

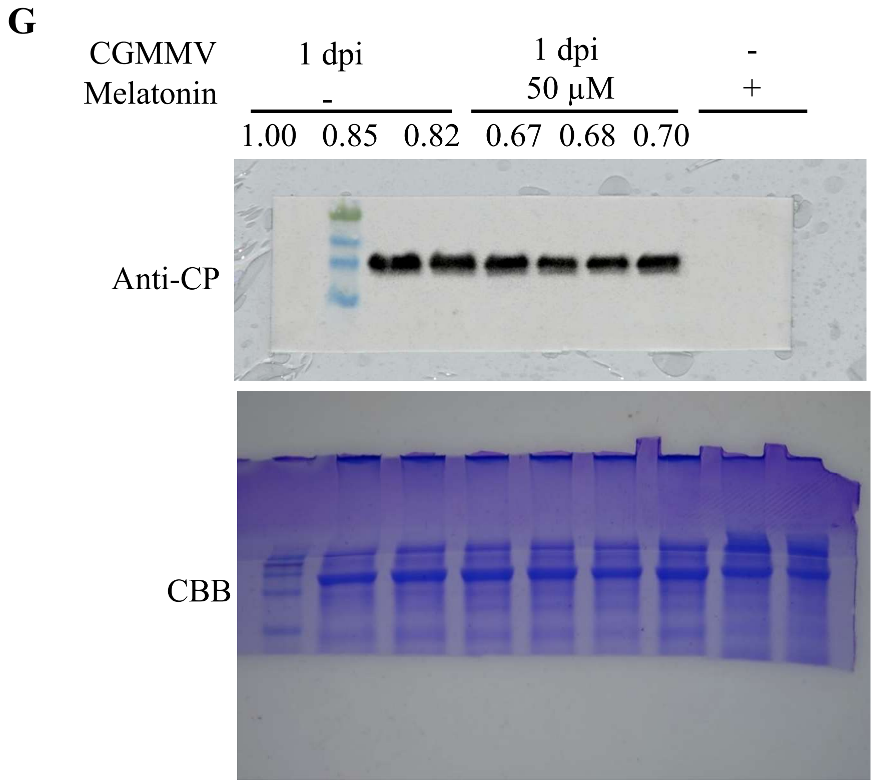

Figure S4

Original picture for Fig.2

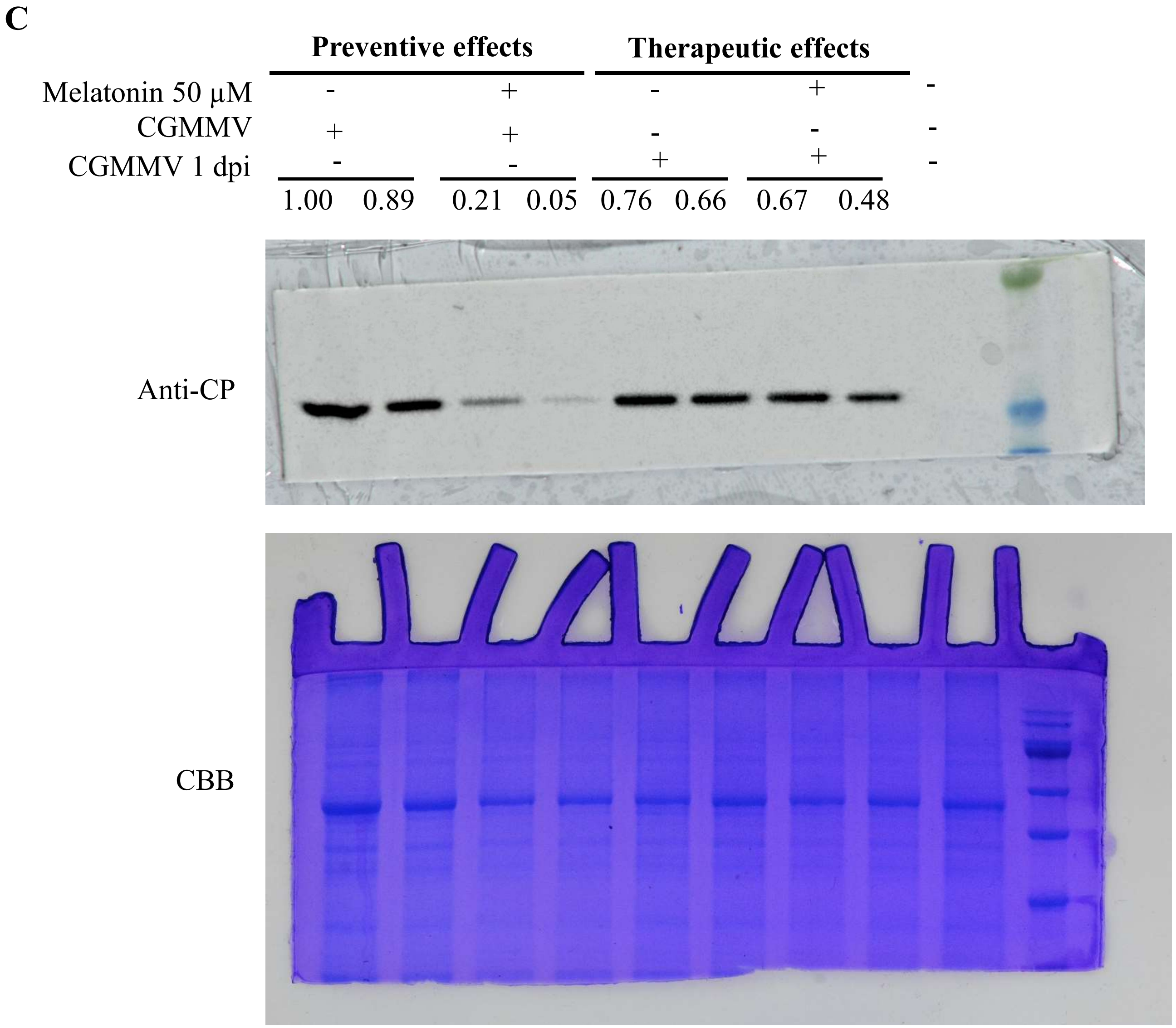

Figure S5

Original picture for Fig.5

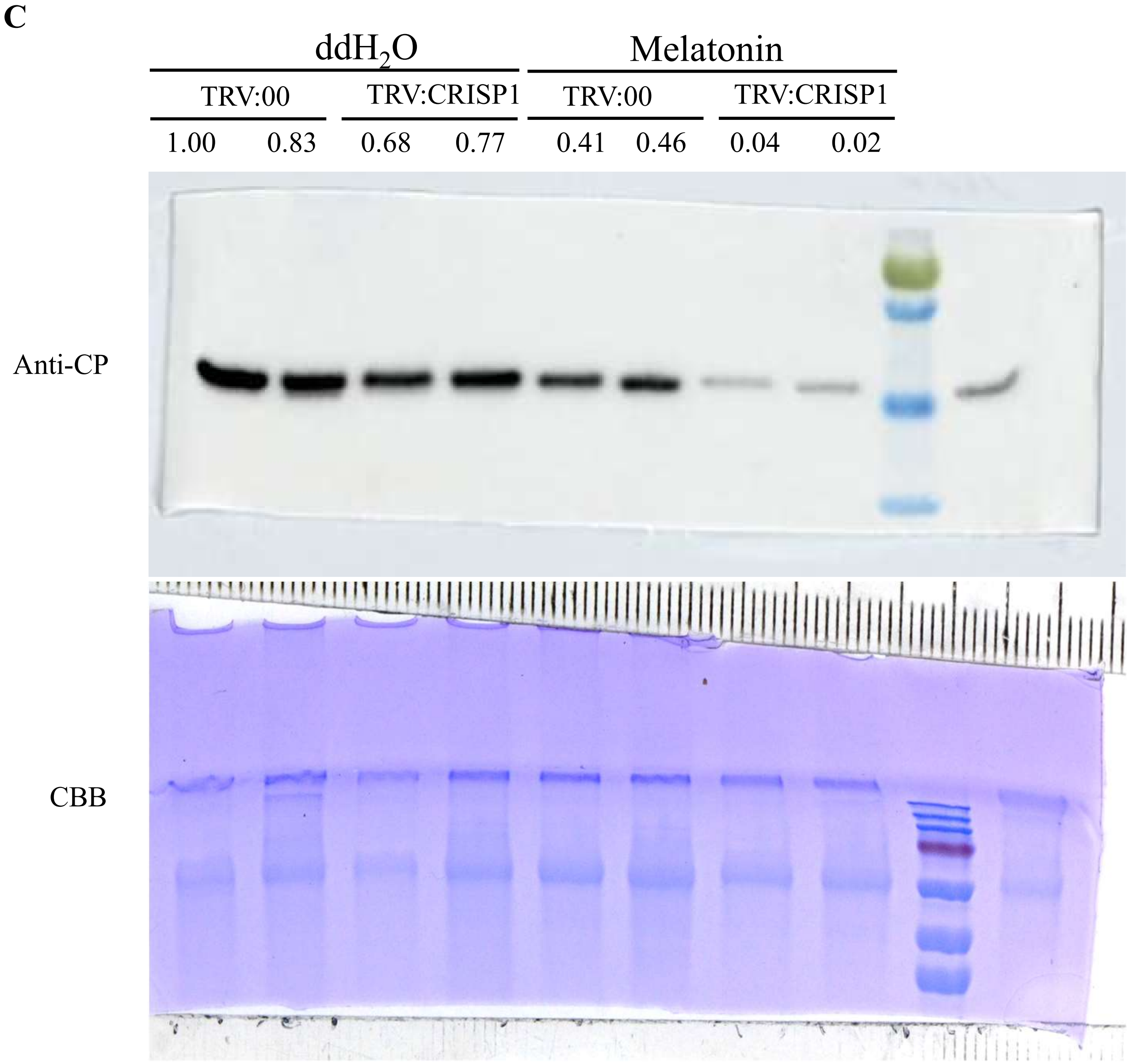

Figure S6

Original picture for Fig.7C

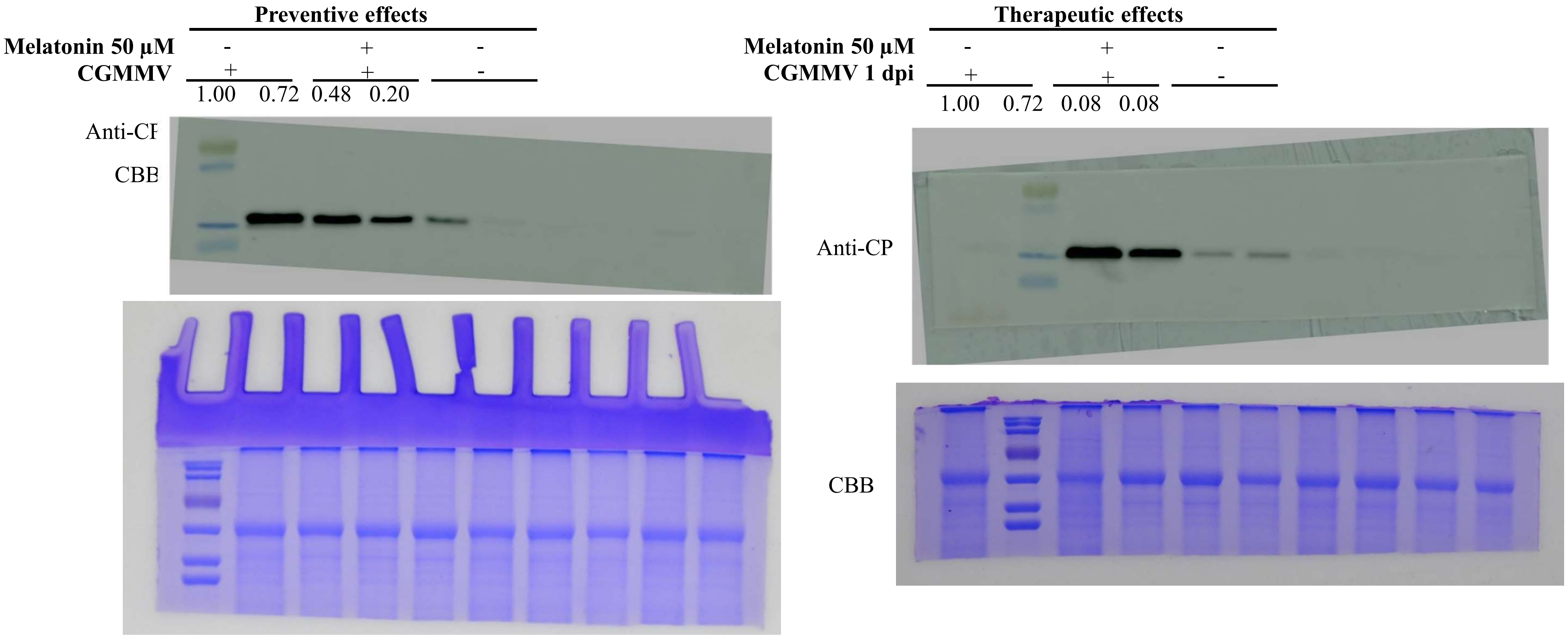

Figure S7

Original picture for Fig.7F

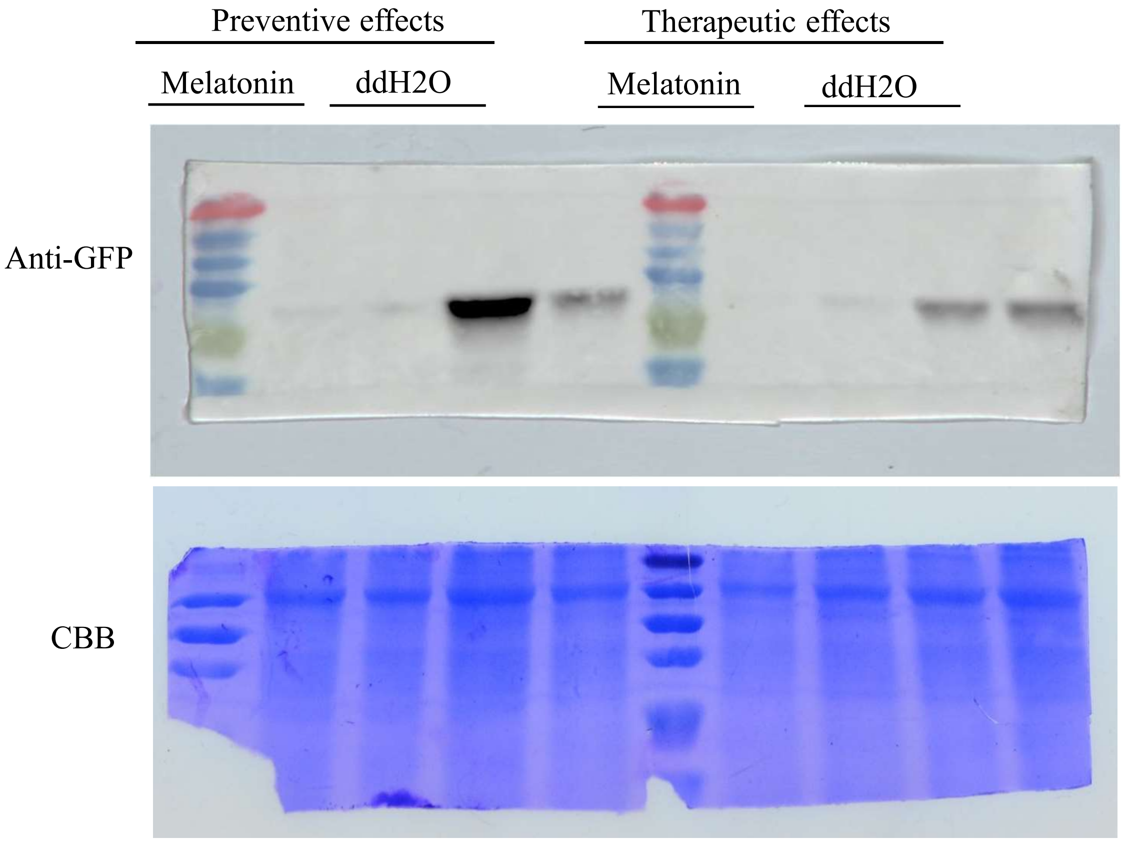

Figure S8
